# L'encéphalopathie postérieure réversible syndrome: à propos d'un cas

**DOI:** 10.11604/pamj.2019.33.154.16630

**Published:** 2019-07-01

**Authors:** Moulay El Mehdi El Hassani, Saad Benali, Jaouad Kouach, Driss Moussaoui Rahali

**Affiliations:** 1 Faculté de Médecine et de Pharmacie de Fès, Fès, Maroc; 2 Service de Gynécologie Obstétrique, Hôpital Militaire d'Instruction Mohamed V, Rabat, Maroc

**Keywords:** Encéphalopathie postérieure réversible, hypertension artérielle, céphalée, imagerie par résonance magnétique, Posterior reversible encephalopathy (PRES), arterial hypertension, headache, magnetic resonance imaging

## Abstract

L'encéphalopathie postérieure réversible (EPR) est une entité radio-clinique associant une atteinte réversible du système nerveux central à une imagerie encéphalique typique. Il existe une grande variabilité dans la présentation clinique de ce syndrome et des aspects en imagerie parfois atypiques. L'EPR est une complication neurologique inhabituelle survenant au cours de la grossesse ou en post-partum, en dehors de toute pathologie préexistante de la grossesse dont l'œdème vasogénique par rupture de la barrière hémato encéphalique paraît être l'acteur principal. Nous rapportons l'observation d'une patiente primipare présentant des crises convulsives généralisées tonico-cloniques associées à un pic hypertensif, survenant au cours du troisième trimestre de grossesse. L'imagerie par résonance magnétique (IRM) cérébrale était en faveur d'une encéphalopathie postérieure réversible. L'EPR doit être évoqué devant tout signe d'appel neurologique, vu l'évolution favorable sans séquelles sous un traitement précoce et rapide.

## Abstract (en)

Posterior reversible encephalopathy syndrome (PRES) is a radioclinical entity associating a reversible central nervous system involvement with a common brain imaging feature. There is a great variability in the clinical presentation of this syndrome and in the features of its sometimes atypical imaging appearance. PRES is an unusual neurological complication occurring during pregnancy or in the post-partum period, other than any pre-existing pathology occurred during pregnancy. Vasogenic edema due to the rupture of the blood-brain barrier seems to be its main cause. We here report the case of a primiparous patient with generalized tonic-clonic seizures associated with hypertensive peak occurred during the third trimester of pregnancy. Magnetic resonance imaging (MRI) of the brain showed posterior reversible encephalopathy. PRES should be suspected in patients with any sign of neurological disorder. It has a favorable outcome without sequelae under early and fast treatment.

## Introduction

L'encéphalopathie postérieure réversible (EPR), est une entité radio-clinique associant une atteinte réversible du système nerveux central à une imagerie encéphalique typique (IRM ou TDM). Il existe une grande variabilité dans la présentation clinique de ce syndrome et des aspects en imagerie parfois atypiques [1, 2]. Elle associe plusieurs signes neurologiques tels que des céphalées [3], des troubles visuels [3], des troubles de la conscience [4], des crises convulsives et des anomalies radiologiques cérébrales bilatérales prédominant dans les régions postérieures qui sont classiquement réversibles[5, 6]. Néanmoins en 2004, Ahn *et al.* ont rapporté une série de 7 patientes présentant des anomalies radiologiques dans d'autres régions définissant cette situation comme « PRES atypiques » [7]. Plusieurs étiologies peuvent être à l'origine de ce syndrome, elles sont dominées par l'encéphalopathie hypertensive [8], la pré-éclampsie, [4, 9, 10], l'éclampsie [10-12], les thérapeutiques immunosuppressives [13, 14], les maladies du système et les atteintes rénales [13, 14]. La recherche d'une pathologie générale sous-jacente devra être systématique, de même que la recherche d'un facteur déclenchant médicamenteux en cas de survenue d'encéphalopathie postérieure réversible [5, 15]. Cette entité est bien décrite dans la littérature, mais encore peu connue par la majorité des cliniciens [5, 16, 17]. L'EPR survient principalement en cas d'hypertension artérielle sévère [18]. Bien que rare, cette affection doit être évoquée devant tout signe d'encéphalopathie survenant dans un contexte d'hypertension artérielle aiguë [19, 20]. Nous rapportons l'observation d'une patiente primipare ayant présenté des crises convulsives généralisées tonico-cloniques associées à un pic hypertensif, avec à l'IRM, des lésions en faveur d'un PRES syndrome.

## Patient et observation

**Figure 1:**
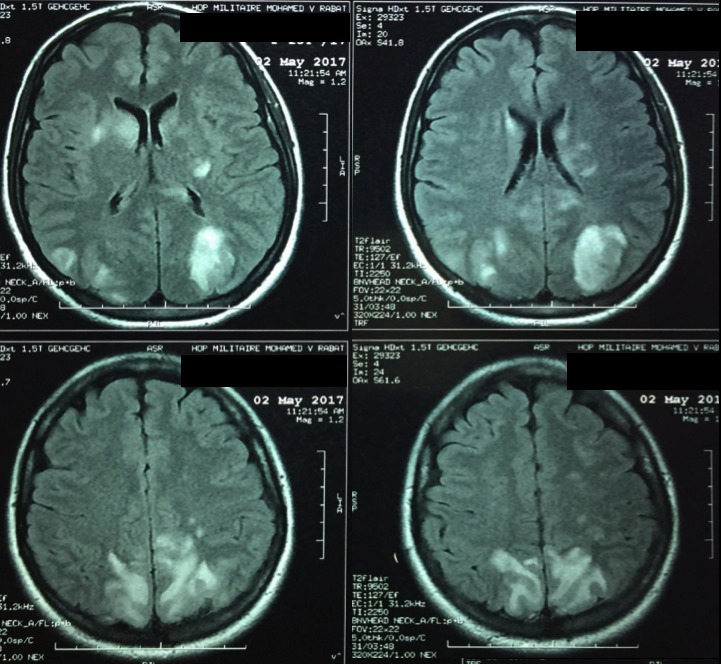
Imagerie par résonance magnétique (IRM) encéphalique, coupe axiale, séquence T2 FLAIR et diffusion: hypersignaux fronto-pariéto-occipitaux bilatéraux, mal systématisés

**Figure 2:**
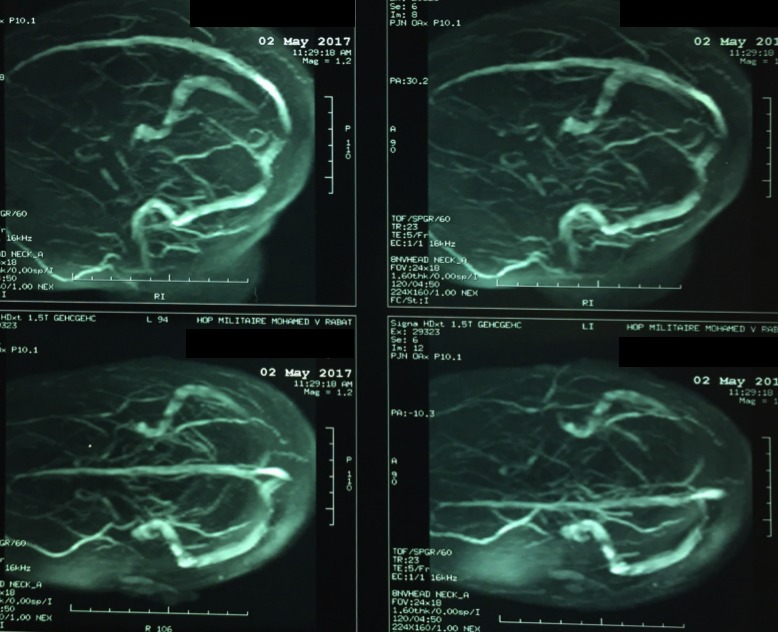
Angio-IRM cétébrale: multiples foyers ischémiques d'origine veineuse

Il s'agit d'une femme âgée de 25 ans, droitière, primipare, sans antécédents pathologiques notables. La patiente a été hospitalisée au service de gynécologie-obstétrique de l'hôpital militaire d'instruction Mohammed V de Rabat, pour prise en charge d'une pré-éclampsie sévère à 27 semaines d'aménorrhée (SA) devant des chiffres tensionnels élevés, une protéinurie positive et des signes neuro-sensoriels à type de céphalées en casque et de troubles visuels à type d'amaurose. La patiente a été mise sous Nicardipine à la seringue auto-pulsée avec surveillance des chiffres tensionnels et de l'état de conscience. L'évolution a été marquée par la survenue de 2 crises convulsives tonico-cloniques généralisées avec retour à l'état de conscience en période intercritique. La patiente a présenté au cours de la crise un pic hypertensif à 180/110 mmHg. Les crises ont cessé sous 10 mg de diazépam (Valium). L'examen après résolution des crises trouvait une pression artérielle à 160/100 mmHg avec retour à l'état de conscience initial. La patiente a été admise en service de réanimation et a été mise sous sulfate de magnésium, sous nicardipine et sous alpha-métyl-dopa avec bonne évolution. Une corticothérapie a été administrée pour maturation pulmonaire fœtale. Au cours de son hospitalisation, la patiente a bénéficié d'une IRM cérébrale objectivant la présence de multiples plages en hypersignal T2 Flair sus tentorielles, mal systématisées, cortico-sous corticales, fronto-pariéto-occipitales bilatérales, des noyaux caudés et des capsules interne et externe. Soit un aspect compatible avec un PRES syndrome dans sa forme atypique. Le bilan biologique comprenant un hémogramme, un ionogramme et un bilan de crase était sans particularités. Un fond d'œil a été demandé revenu normal. La patiente a bénéficié d'une extraction par césarienne à 28 SA et l'évolution en post-partum était sans particularités. La patiente est sortie de l'hôpital et aucune récidive n'était observée. Elle a bénéficié d'une IRM de contrôle 3 mois plus tard ayant objectivé la disparition de toutes les lésions (Figure 1, Figure 2).

## Discussion

En 2006, Narbone *et al.* ont noté que les anomalies radiologiques du cerveau ne sont pas exclusivement postérieures et que la réversibilité des lésions dépend de la sévérité de celles-ci et d'une prise en charge thérapeutique rapide et adéquate [21].

**Aspects épidémiologiques**: l'encéphalopathie postérieure réversible est une pathologie rare, peu connue et reste probablement sous-diagnostiquée [22]. L'incidence mondiale de l'EPR est inconnue [23]. Environ 60% des cas d'encéphalopathie postérieure réversible sont secondaires, le plus souvent au post-partum. Elle peut néanmoins survenir en fin de grossesse. Cependant, indépendamment de la grossesse, certains traitements hormonaux modifiant les taux d'œstrogène et de progestérone ont été incriminés dans la survenue d'EPR [24].

**Aspects physiopathologiques**: cette affection se caractérise par une vasoconstriction prolongée mais spontanément réversible des artères cérébrales, survenant de façon spontanée ou dans des circonstances particulières [6, 25]. deux théories restent les plus acceptées [18, 26-28].

**La première théorie**: elle est basée sur la séquence hypertension-dépassement de l'autorégulation cérébrale-hyperperfusion-œdème cérébral vasogénique par fuite liquidienne extracapillaire. L'innervation sympathique périvasculaire exerçant un effet protecteur est plus riche dans la circulation antérieure et le système carotidien, d'où la prédominance postérieure des lésions.

**La deuxième théorie**: elle est en faveur d'une vasoconstriction cérébrale secondaire à l'hypertension artérielle ou à un processus systémique. Ce phénomène d'autorégulation entraînerait une baisse de la perfusion cérébrale et donc un œdème cytotoxique. Cette théorie est appuyée par l'absence d'élévation tensionnelle (ou minime) dans certains cas. Elle plaide plus en faveur d'un processus systémique (infection, pré-éclampsie, transplantation, chimiothérapie anticancéreuse) responsable d'une activation du système immunitaire et des cellules endothéliales suivie d'une altération endothéliale avec hypoperfusion secondaire (systémique ou régionale).

### Aspects cliniques

**Facteurs favorisants**: les circonstances favorisant la survenue de l'EPR sont nombreuses. Les plus fréquentes sont: 1) l'hypertension artérielle [15]: elle est classique et a été le premier facteur décrit (encéphalopathie hypertensive). Une HTA modérée à sévère est observée chez 75% des patientes; 2) l'éclampsie [18] et la pré-éclampsie [11]: le lien a été souvent établi, y compris avec une tension artérielle normale. Des survenues tardives jusqu'à plusieurs semaines après l'accouchement ont été rapportées; 3) les chimiothérapies telles que le cyclophosphamide peuvent induire une EPR même en l'absence d'autres facteurs de risque connus; 4) l'insuffisance rénale chronique et la dialyse; 5) les maladies auto-immunes.

**Manifestations cliniques**: les manifestations cliniques sont variées [12]. Elles associent de manière variable quatre symptômes neurologiques cardinaux: les céphalées, les crises convulsives, les troubles de la conscience et les troubles visuels, ces signes sont accompagnés le plus souvent d'une augmentation aiguë et brutale de la pression artérielle [25].

**Les céphalées**: elles sont sévères chez 88 à 100% des patientes, le plus souvent « en coup de tonnerre ». La céphalée est bilatérale, à début postérieur devenant diffuse volontiers décrite comme « la pire céphalée jamais ressentie ». La présence de cervicalgies latérales ou postérieures doit faire rechercher une dissection vertébrale ou carotidienne associée.

**Les crises convulsives**: les déficits et les crises convulsives peuvent être de tout type. Certains déficits transitoires sont d'installation brutale compatibles avec un accident ischémique transitoire, alors que d'autres s'installent progressivement sur plusieurs minutes, accompagnés de phénomènes positifs visuels et/ou sensitifs et d'une marche migraineuse, évoquant des auras symptomatiques.

**Les poussées hypertensives**: elles surviennent au cours des accès céphalalgiques. Ainsi, un tiers des patientes environ a une pression artérielle élevée lors des épisodes douloureux [6].

**Les troubles visuels**: ils sont présents dans plus de 50% des cas [29]. On retrouve de nombreux signes tels qu'une vision floue un scotome scintillant, une négligence visuelle [6], une hémianopsie, une photophobie ou une cécité corticale.

**Les troubles de conscience**: ils peuvent exister mais sont généralement discrets. Cependant, un coma est possible dans les rares formes catastrophiques avec hématomes et/ou des infarctus multiples [3].

### Aspects paracliniques

**Examens radiologiques**: l'encéphalopathie postérieure réversible se manifeste sur le plan radiologique par des anomalies de la substance blanche et de la substance grise [12] touchant préférentiellement les régions postérieures suggérant un œdème des régions cérébrales postérieures pariéto-occipitales (Tableau 1) [30]. L'imagerie est indispensable et doit être effectuée dans les délais les plus brefs pour permettre un diagnostic précoce afin d'instaurer un traitement adéquat et limiter le risque de lésions irréversibles.

**Tableau 1:** Localisations des différentes atteintes de l’EPR

Localisations	Prevalence (%)
Lobes occipitaux	100
Lobes pariétaux	59
Lobes frontaux	30
Lobes temporaux	13
Cervelet	12
Corps calleux	6
Thalamus	5
Tronc cerebral	3

**La tomodensitométrie (TDM) cérébrale**: la tomodensitométrie (TDM) est fréquemment anormale, avec des hypodensités postérieures diffuses ne prenant pas le contraste [1]. À la phase initiale, le scanner n'est pas un examen performant pour le diagnostic car en l'absence de complications hémorragiques ou ischémiques du parenchyme, il est faussement rassurant en étant normal dans 40% des cas.

**L'imagerie par résonance magnétique (IRM)**: elle est souvent caractéristique ce qui permet de poser le diagnostic dans un contexte clinique évocateur mais peut être également normale. Bien que les lésions de l'encéphalopathie postérieure puissent être détectées par la TDM cérébrale, l'IRM est considérée comme l'examen de référence. L'IRM cérébrale comprend plusieurs séquences: 1) les lésions apparaissent en isosignal ou hyposignal T1 et hypersignal T2 et FLAIR. Il n'y a habituellement pas de rehaussement après injection de produit de contraste [11]. 2) l'IRM en séquence de diffusion est le meilleur outil diagnostique permettant un traitement adapté et rapide afin de prévenir l'apparition de lésions neurologiques irréversibles et de séquelles permanentes. Les lésions sont souvent sous-corticales, bilatérales et symétriques dans les régions pariéto-occipitales. Des lésions corticales sont possibles, mais rares [27]. L'atteinte du tronc cérébral et du cervelet est fréquente, alors que l'atteinte du lobe frontal est rare et souvent associée à un pronostic péjoratif. L'aspect typique montre des lésions diffuses sous-corticales et profondes.

**L'angiographie ou artériographie cérébrale**: elle met en évidence des irrégularités vasculaires avec des vasoconstrictions focales et diffuses et des vasodilatations focales souvent responsables d'un aspect en « collier de perle » même en l'absence d'une hypertension artérielle significative.

**Autres techniques d'imagerie**: d'autres techniques d'imagerie cérébrale peuvent être utilisées telles que la spectroscopie par résonance magnétique à proton, la cartographie ADC et l'angio-IRM 3D. Le gold standard reste l'angiographie; elle est l'examen le plus sensible, mais peut être normale lorsqu'elle est faite précocement. Le diagnostic de l'EPR, comme son nom l'indique, ne peut être posé qu'après vérification de la réversibilité par une imagerie vasculaire à trois mois (TDM, ARM ou artériographie) montrant un retour à la normale, ou du moins une nette régression de l'atteinte vasculaire.

**Examens biologiques**: les examens biologiques ne sont pas contributifs dans le diagnostic positif de l'EPR. La ponction lombaire est de réalisation quasiment systématique, afin d'éliminer une hémorragie méningée si le scanner cérébral est normal.

### Autres examens

**Electroencéphalogramme**: en général, l'électroencéphalogramme montre des tracés lents et peu réactifs sans signes de focalisation.

### Aspects thérapeutiques et évolutifs

**Aspects therapeutiques**: la stratégie thérapeutique de l'EPR dépend de son étiologie et de son tableau clinique. L'arrêt du facteur déclenchant ou aggravant représente la première mesure thérapeutique [31]. Il n'existe pas de prise en charge thérapeutique standardisée. De nombreux traitements ont été décrits avec des résultats discordants. Cependant le contrôle de l'HTA est le volet primordial du traitement. Il fait appel aux agents antihypertenseurs habituels: inhibiteurs calciques (nicardipine ou diltiazem), bétabloquants (labetolol notamment) et diurétiques. L'objectif thérapeutique est de maintenir une pression artérielle moyenne entre 105 et 125 mmHg, sans réduire cette pression de plus de 25% durant la première heure. Le sulfate de magnésium possède un effet vasodilatateur, qui augmente le flux sanguin cérébral évitant ainsi l'apparition de lésions ischémiques [6, 9] qui sont à l'origine de crises convulsives. Les corticoïdes sont les médicaments les plus utilisés pour lutter contre le vasospasme et les céphalées. L'association avec un traitement anti-œdémateux, le mannitol en l'occurrence doit être discutée au cas par cas et ne peut être bénéfique que dans certaines situations [31]. La régression spontanée de l'EPR rend cependant difficile l'appréciation de l'efficacité de ces traitements. Par ailleurs, le traitement de l'hypertension artérielle doit être prudent car il convient de ne pas induire d'hypotension alors qu'il existe déjà un vasospasme cérébral réduisant le débit cérébral. En cas de crise convulsive, un traitement antiépileptique doit être instauré en urgence. Les benzodiazépines doivent être administrés en première ligne par voie intraveineuse [13].

**Aspects évolutifs**: le diagnostic et le traitement adapté et rapide de l'EPR permettent de prévenir l'apparition de lésions neurologiques irréversibles et de séquelles permanentes. Malgré la sévérité du tableau clinique initial (coma, état de mal épileptique), pouvant nécessiter une prise en charge intensive, l'évolution de l'EPR est généralement favorable, sous réserve d'une prise en charge précoce et adaptée. On observe le plus souvent une régression complète des anomalies clinico-radiologiques en quelques semaines. Dans 90% des cas, les manifestations neurologiques régressent dès le septième jour après le début du traitement antihypertenseur et/ou étiologique. Une amélioration radiologique est de règle en quinze jours, néanmoins une normalisation n'est parfois obtenue qu'au-delà d'une année. Il faudrait souligner que l'absence de prise en charge thérapeutique précoce peut entraîner une aggravation clinique ou des séquelles à type de crises épileptiques ou de déficits neurologiques [8]. Plusieurs cas de décès au cours de l'EPR ont été rapportés ce qui a même poussé certains auteurs à proposer le terme d'encéphalopathie potentiellement réversible [23]. L'EPR, en raison de sa description relativement récente, n'a pas encore fait l'objet d'études longitudinales prolongées, l'évolution clinique des patientes après la régression du premier épisode est de ce fait très mal connue. Le diagnostic de l'EPR, comme son nom l'indique, ne peut être posé qu'après vérification de la réversibilité par une imagerie vasculaire à trois mois (TDM, ARM ou artériographie) montrant un retour à la normale, ou du moins une nette régression de l'atteinte vasculaire.

## Conclusion

L'EPR est une complication neurologique inhabituelle, en dehors de toute pathologie préexistante de la grossesse [12] . C'est un syndrome encore méconnu, bien que relativement fréquent. Son pronostic peut être effroyable lorsqu'il n'est pas reconnu et pris en charge à temps. Il est donc fondamental d'y être sensibilisé et de faire preuve de vigilance afin de poser le diagnostic précocement, et d'instaurer une prise en charge efficace en situation d'urgence obstétricale [27]. La recherche d'une pathologie générale sous-jacente devra être systématique, de même que la recherche d'un facteur déclenchant médicamenteux. Le diagnostic et le traitement adapté et rapide de l'EPR [100] permettent de prévenir l'apparition de lésions neurologiques irréversibles et de séquelles permanentes.

## Conflits d’intérêts

Les auteurs ne déclarent aucun conflit d'intérêts.
